# Anti-Inflammatory and Anticancer Effects of Anthocyanins in In Vitro and In Vivo Studies

**DOI:** 10.3390/antiox13091143

**Published:** 2024-09-22

**Authors:** Tomasz Kowalczyk, Martyna Muskała, Anna Merecz-Sadowska, Joanna Sikora, Laurent Picot, Przemysław Sitarek

**Affiliations:** 1 Department of Molecular Biotechnology and Genetics, Faculty of Biology and Environmental Protection, University of Lodz, Banacha 12/16, 90-237 Lodz, Poland; tomasz.kowalczyk@biol.uni.lodz.pl; 2 Students Research Group, Department of Medical Biology, Medical University of Lodz, Muszynskiego 1, 90-151 Lodz, Poland; martyna.muskala@student.umed.lodz.pl; 3 Department of Allergology and Respiratory Rehabilitation, Medical University of Lodz, 90-725 Lodz, Poland; anna.merecz-sadowska@uni.lodz.pl; 4 Department of Bioinorganic Chemistry, Medical University of Lodz, Muszynskiego 1, 90-151 Lodz, Poland; joanna.sikora@umed.lodz.pl; 5 Littoral Environnement et Sociétés UMRi CNRS 7266 LIENSs, La Rochelle Université, 17042 La Rochelle, France; laurent.picot@univ-lr.fr; 6 Department of Medical Biology, Medical University of Lodz, Muszynskiego 1, 90-151 Lodz, Poland

**Keywords:** anthocyanins, flavonoids, antioxidants, anti-inflammatory, anticancer, chronic diseases, in vitro studies, in vivo studies, bioavailability

## Abstract

Anthocyanins, a class of flavonoid compounds responsible for the vibrant colors of many fruits and vegetables, have received considerable attention in recent years due to their potential health benefits. This review, focusing on evidence from both in vitro and in vivo studies, provides a comprehensive overview of the current state of knowledge regarding the health-promoting properties of anthocyanins. The chemical structure and diversity of anthocyanins, their bioavailability, and their mechanisms of action at the cellular and molecular level are examined. Research on the antioxidant, anti-inflammatory, anticancer, and neuroprotective effects of anthocyanins is critically reviewed. Special emphasis is placed on the role of anthocyanins in the prevention and treatment of chronic diseases such as cardiovascular diseases, diabetes, and neurodegenerative diseases. This review also discusses the challenges of translating in vitro findings to in vivo and highlights the importance of considering dose, bioavailability, and metabolism when assessing the therapeutic potential of anthocyanins. This review concludes with the identification of gaps in current research and suggestions for future directions for anthocyanin studies, including the need for more long-term clinical trials and investigations into potential synergistic effects with other phytochemicals. This comprehensive analysis highlights the promising role of anthocyanins in promoting human health and provides valuable insights for researchers, health professionals, and the nutraceutical industry. This study provides new insights, as it comprehensively investigates the dual anti-inflammatory and anticancer effects of anthocyanins in both in vitro and in vivo models. By uncovering the biological properties of anthocyanins from a variety of natural sources, this research not only expands our knowledge of the action of these compounds at the cellular level, but also enhances their clinical relevance through in vivo validation. Furthermore, the innovative use of anthocyanins may lead to important advances in their therapeutic application in the future.

## 1. Introduction

Plant-derived bioactive compounds represent a vast and diverse group of molecules that have evolved over millions of years as part of plants’ adaptive strategies. These compounds, while not essential for basic plant metabolism, play critical roles in plant survival, reproduction, and environmental interactions. The biosynthesis of these secondary metabolites is often triggered by specific environmental stresses or developmental stages, leading to a complex and dynamic phytochemical profile within each plant species [1,2]. This natural diversity offers an extensive reservoir of potentially beneficial compounds for human use. The study of plant bioactive compoun encompasses not only their identification and characterization but also the investigation of their biosynthetic pathways, genetic regulation, and ecological functions. Understanding these aspects can lead to the development of enhanced cultivation practices, breeding programs, and biotechnological approaches to optimize the production of desired compounds. In addition, research into traditional medicinal plants continues to uncover new bioactive compounds, combining ethnobotanical knowledge with modern scientific research [3,4].

For centuries, plants have been the primary source of chemical compounds with various health-promoting properties [5]. They are called phytochemicals. Substances contained in plants play a key role in the prevention and treatment of numerous diseases. Initially, their properties were studied organoleptically through experiments conducted in folk medicine. Over the years, and with the progress of science, it became possible to conduct research on specific substances contained in, among others, extracts or essential oils of plant origin. Chemical knowledge was also deepened, allowing these key compounds to be divided into classes. The most important groups of compounds contained in plants include polyphenols [6], carotenoids [7], glucosinolates, saponins, phytosterols, and terpenoids [8]. Each of these groups has unique properties that contribute to the improvement of human health.

**Figure 1:**
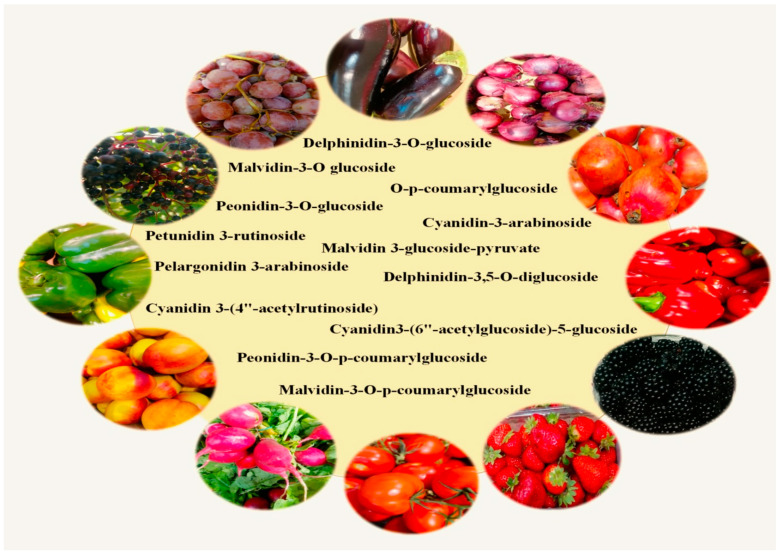
Natural anthocyanins and their sources in the diet.

This paper will be mainly devoted to anthocyanins—a group of natural plant dyes that belong to the flavonoid family. Anthocyanins (from the Greek *anthos* = flower and *kianos* = blue) [9] are found in many fruits, vegetables, flowers, and leaves, giving them characteristic red, purple, and blue colors (Figure 1). These pigments occur in different combinations and proportions, which affects the richness of the colors [10]. Anthocyanin pigments are easily degraded during food processing and storage. This affects the color, quality, and nutritional properties [11]. At low pH (acidic conditions), anthocyanins are more stable, resulting in a red pigment, while higher pH values will cause the blue color to fade [12]. It should be emphasized that acetylation and glycosylation significantly affect the biological function of anthocyanins, increasing, among other things, their stability, solubility, and bioavailability. Acetylation increases the stability of anthocyanins, especially against environmental factors such as heat, light, and pH changes. This makes them more effective as pigments and more resistant to degradation, potentially prolonging their biological activity. Glycosylation increases the solubility and bioavailability of anthocyanins. It also influences the way they are absorbed, transported, and metabolized in the body, affecting their antioxidant activity and potential health benefits. In conclusion, both modifications enhance the functional and biological properties of anthocyanins, improving their anticancer and antioxidant properties. In recent years, there has been a growing interest in the health-promoting properties of anthocyanins, which is due to both their potential health benefits and growing awareness of the importance of a healthy diet. The introduction of anthocyanins into the daily diet can have a number of benefits for human health. Numerous studies have suggested that anthocyanins have powerful antioxidant properties, which means that they can neutralize harmful free radicals in the body and protect cells from oxidative damage [13,14,15,16]. In addition, anthocyanins have anti-inflammatory effects [13,17,18,19], which may be important in preventing and treating chronic inflammatory diseases. These compounds can also be used to help treat conditions such as heart disease [20,21,22], type 2 diabetes [19,23,24,25], and some cancers [26]. Anthocyanins also have beneficial effects on eye health [27,28], especially considering the complications resulting from elevated blood glucose levels—diabetic retinopathy and prevention of macular degeneration in eye-related diseases. Additionally, studies indicate that consuming foods rich in anthocyanins can support cognitive function, improve memory, and also have a supportive effect on some neurodegenerative diseases, which is particularly important in the context of an aging society [29,30,31,32,33].

This paper will discuss the main sources of anthocyanins, some mechanisms of their health-promoting effects with specific examples, and the results of the latest scientific research on their impact on human health. We will also present an analysis of the potential applications of anthocyanins in the prevention and therapy of various diseases, as well as the prospects for further research in this field. We will mainly emphasize the anti-inflammatory and anticancer effects of anthocyanins in scientific works, both in vitro and in vivo.

## 2. Study Design

**Figure 2:**
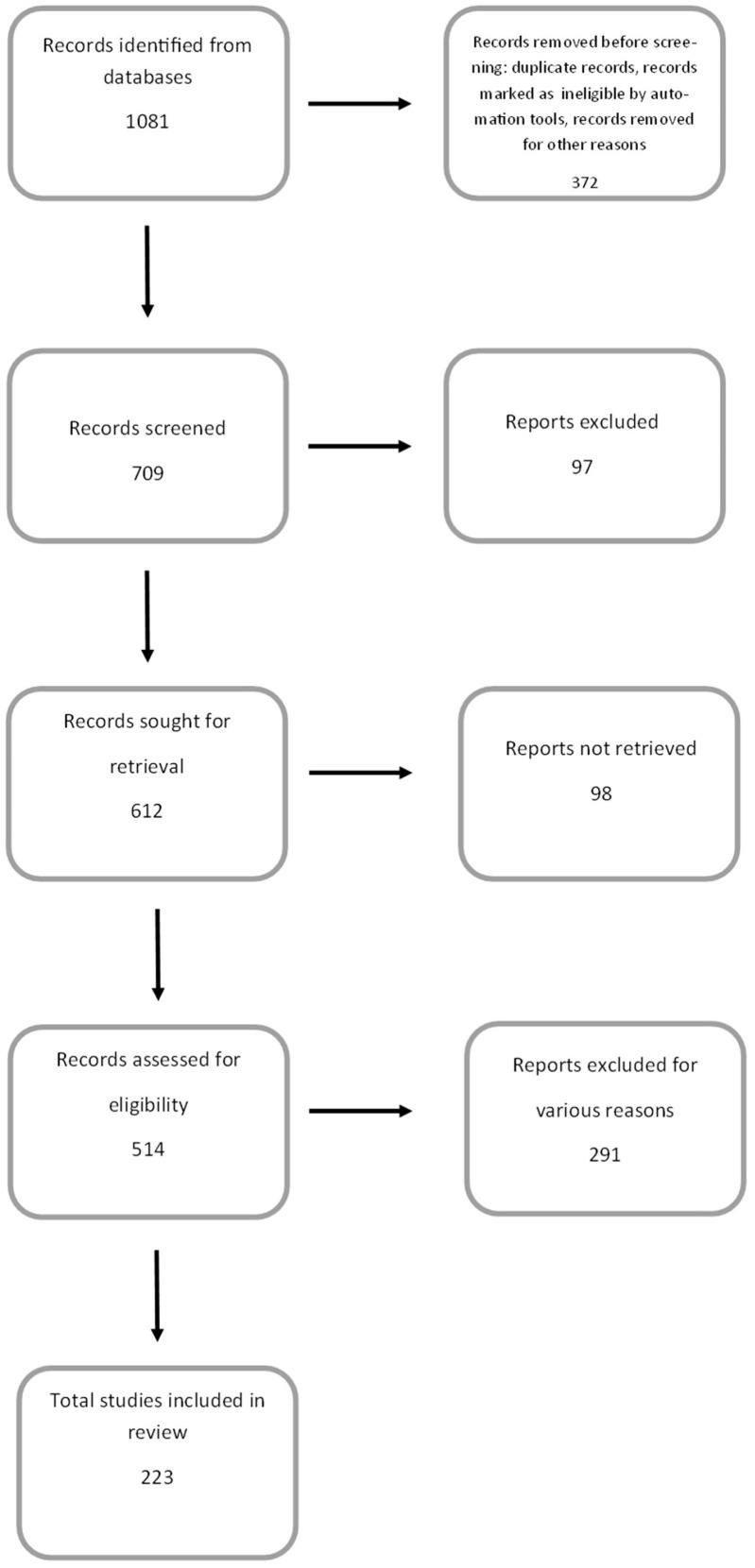
PRISMA flow diagram demonstrating the screening method for the article.

A search of appropriate literature, i.e., health benefits of anthocyanins in in vitro and in vivo studies, was performed using Google Scholar and NCBI-PubMed. The following keywords were used: anthocyanins, anthocyanidins, cyanidin, delphinidin, peonidin, petunidin, malvidin, anti-inflammatory/anticancer/antiproliferative/antioxidant/cardioprotective/neuroprotective/ properties of anthocyanins, in vitro models, and in vivo models. While the search term “health benefits of anthocyanins” identified more than a thousand articles in the PubMed database dating back to 1920, this review primarily included articles from 2000 onwards. These accounted for 94.6% of all articles used in this review. During the course of the work, some articles were rejected due to their inconsistency with the topic, lack of reliable data, or the presence of general information that did not specify the relationship being studied (Figure 2).

### Chemical Structure of Anthocyanins

**Figure 3:**
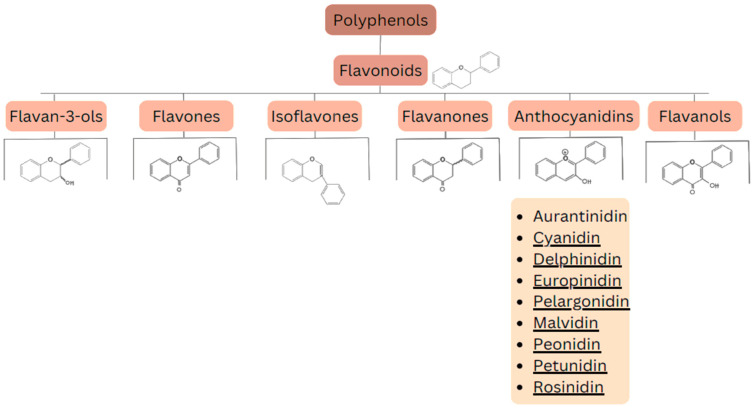
Division of polyphenols into different classes of compounds.

Flavonoids are a group of naturally occurring chemical compounds found in plants [34]. They are polyphenols that play an important role in defending plants against pathogens [35] and UV radiation [36,37,38] (this protective effect has also been analyzed for the prevention and treatment of skin disorders). Flavonoids also have numerous health benefits for humans, including antioxidant effects: they neutralize free radicals [39], which can help prevent cell damage; anti-inflammatory effects [40]: they can reduce inflammation in the body; anticancer effects: research suggests that flavonoids may inhibit growth of cancer cells [41]; and cardioprotective effects: they can improve the function of blood vessels and lower blood pressure [42,43]. Flavonoids can be divided into several main groups, including: flavan-3-ols (catechins) (e.g., epicatechin, epigallocatechin), flavones (e.g., apigenin, luteolin), isoflavones (e.g., genistein, daidzein), flavanones (e.g., naringenin, hesperidin), anthocyanins (e.g., cyanidin, delphinidin), and flavonols (e.g., quercetin, kaempferol). The division of these compounds is presented in Figure 3.

A very interesting group among the above-mentioned is the anthocyanins. Anthocyanins are a group of flavonoids responsible for the red, purple, and blue colors of many fruits, vegetables, and flowers [44]. They have a wide range of health benefits and play an important role in protecting plants from harmful environmental factors. These natural pigments of plant origin are found in vacuoles (mainly in flowers and fruits) in the form of granules of various sizes around 3 to 10 μm in diameter [45]. Anthocyanins are anthocyanidin glycosides, which means that they consist of an aglycone (anthocyanidin) molecule connected to a sugar molecule [46]. Their chemical structure is crucial for their color and biological properties.

Anthocyanins can be divided into their three most important parts:

1. **Anthocyanidins**: Aglycones, which are the basic chemical structure of anthocyanins. They have a ion flavylium structure (2-phenylchromene), which is responsible for their color. It contains seven different chemical side groups, which can be composed of hydrogen atoms, hydroxyl groups, and methoxy groups. The most common anthocyanidins are cyanidin, delphinidin, pelargonidin, peonidin, petunidin, and malvidin.
2. **Sugar part**: Anthocyanidins are linked to one or more sugars through glycosidic bonds. The most common sugars are glucose, galactose, and rhamnose. This combination increases the solubility of anthocyanins in water and the stability of the dye [47].
3. **Structural modifications**: Anthocyanins can have various functional groups, such as hydroxyl (-OH) and methoxyl (-OCH3) groups, which influence their chemical properties and color [10]. These modifications can affect the intensity and range of the light absorption spectrum, which is responsible for the variety of colors they can take on.

**Figure 4:**
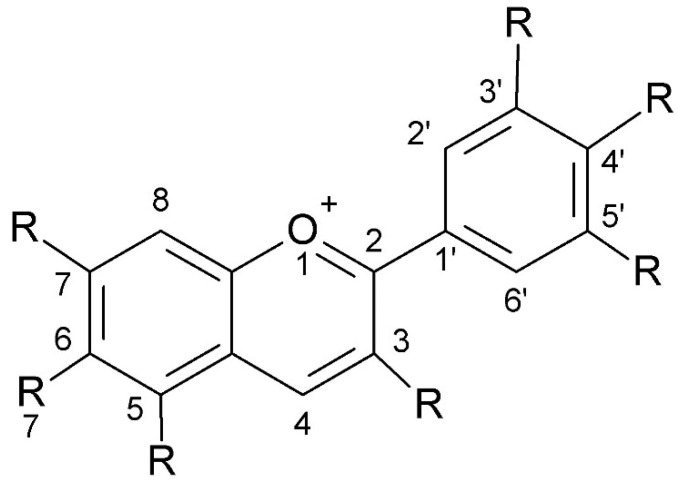
The chemical structure of anthocyanins (created with ChemSketch).

Anthocyanins have a complex chemical structure consisting of three rings: two aromatic rings (A and B) and one heterocyclic ring with oxygen (C) of cationic nature, often occurring as flavylium (2-phenylchromene). The C15 skeleton is based on a chromate ring bearing a second aromatic ring B in position 2 (C6-C3-C6). The parent structure of anthocyanidins is the flavylium cation [48]. The model is shown in Figure 4.

High anthocyanin content is found in blueberries, raspberries, black currants, elderberries, blackberries, chokeberries, red cabbage, grapes, and eggplants [49,50,51,52,53,54,55,56]. For industrial purposes, anthocyanins are obtained by extraction from red cabbage leaves or grape skins [57,58]. Some species and examples of anthocyanins found in them are presented in Table 1.

## 3. Anthocyanins and Their Biological Activity

### 3.1. Cyanidin

**Figure 5:**
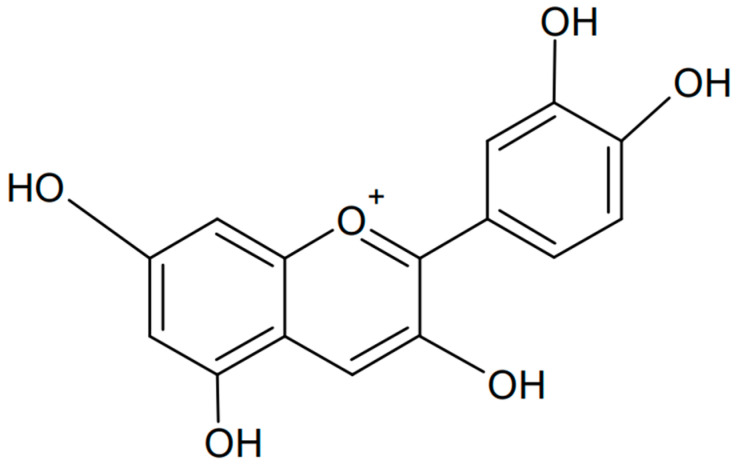
Chemical formula of cyanidin (created with ChemSketch).

Cyanidin is one of the anthocyanins that has been most extensively investigated. It has a flavanol-based chemical structure with hydroxyl and methoxyl groups. Chemically, cyanidin is known as the 3,3′,4′,5,7-pentahydroxyflavyl cation. The formula of cyanidin is presented in Figure 5. This organic compound is found in the largest amounts in the skin of fruits, especially red ones such as apples, plums, and hawthorn [64,65,66]. Its color depends on the pH value: it appears red in acidic conditions and blue in alkaline conditions.

Cyanidin has many health benefits, including its use in the treatment of pathologies associated with reactive oxygen species and their neutralization. Cyanidin and cyanidin 3-*O*-β-D-glucoside have shown a protective effect on DNA cleavage and a dose-dependent activity of scavenging free radicals. In one study, the inhibition of xanthine oxidase (XO) activity was also examined [67]. Anthocyanins are powerful antioxidants that neutralize free radicals and protect cells from oxidative damage. Cyanidin has better antioxidant properties than even vitamin E [68]. One study on pigmented oranges showed that cyanidin-3-O-β-glucopyranoside should be considered one of the most effective antioxidants in dietary plants [69]. Cyanidin glucoside is a highly efficient scavenger of free oxygen radicals in direct interaction with reactive oxygen species (H_2_O_2_, -O_2_, OH·).

The effect of cyanidin on the oxidation of low-density lipoproteins (LDLs) is important in the pathogenesis of atherosclerosis. Studies suggest that anthocyanins may have anticancer properties, helping to prevent the development of cancer. This is supported by the work of Feng et al., who studied the effect of cyanidin-3-rutinoside in several leukemia and lymphoma cell lines. Cyanidin induced the accumulation of superoxides, which are involved in the induction of apoptosis in HL-60 cells. Furthermore, the activation of p38 MAPK and JNK contributed to cell death via activation of the mitochondrial pathway, mediated by Bim [70]. An antitumor effect was also confirmed in lung large-cell carcinoma in nude mice [71]. The study showed impaired tumor growth, increased tumor apoptosis, reduced levels of inflammatory cytokines (IL-1β, TNF-α, C-reactive protein, and IL-6), reduced factors associated with inflammation (cyclooxygenase-2 protein and nuclear factor-κB (NF-κB) mRNA), increased inhibition of NF-κB kinase mRNA, and downregulation of metastasis-related factors (transforming growth factor-β, CD44, epidermal growth factor receptor, and vascular endothelial growth factor) after cyanidin-3-*O*-glucoside treatment. Studies have also confirmed inhibition of the proliferation of B16-F10 murine melanoma cells. The results suggest that anthocyanins derived from elderberry may be used as local adjuvant agents in skin cancer therapy [72]. Caco-2 cells in vitro were the next line tested for responses after cyanidin-3-*O*-glucoside administration [73]. This confirmed inhibition of the proliferation of human tumor cells. Cyanidin-glucoside also modulated cell cycle progression in a liver precancerous lesion in vivo study and additionally influenced the induction of apoptosis in prostate cancer [74], in the induction of apoptosis in colorectal cancer cells via the NF-κB signaling pathway [75], and in renal cell carcinoma tumorigenesis [76]. An extremely important and relatively newly discovered correlation is between anthocyanin consumption and eye health. Inhibitors of diabetic cataracts were studied among anthocyanin monomers isolated from grape skin extract. Using rat lens organ cultures, malvidin 3-glucoside, delphinidin 3-glucoside, cyanidin 3-glucoside, petunidin 3-glucoside, and peonidin 3-glucoside showed an inhibitory effect on lens opacity [77]. Another disease entity in which anthocyanins have been successfully used is the inhibition of the dangerous consequences of diabetes, specifically diabetic retinopathy [25]. Anthocyanins can support heart health by lowering blood pressure, improving blood vessel function, and reducing the risk of atherosclerosis. The results showed that cyanidin glucosides reduced the infarct area, alleviated pathological changes, inhibited ST-segment elevation, and attenuated oxidative stress, ferroptosis-related protein expression, USP19, Beclin1, NCOA4, and LC3II/LC3I expression. The compound could potentially be used as a myocardial protection agent against ischemia-induced damage [78].

### 3.2. Peonidin

**Figure 6:**
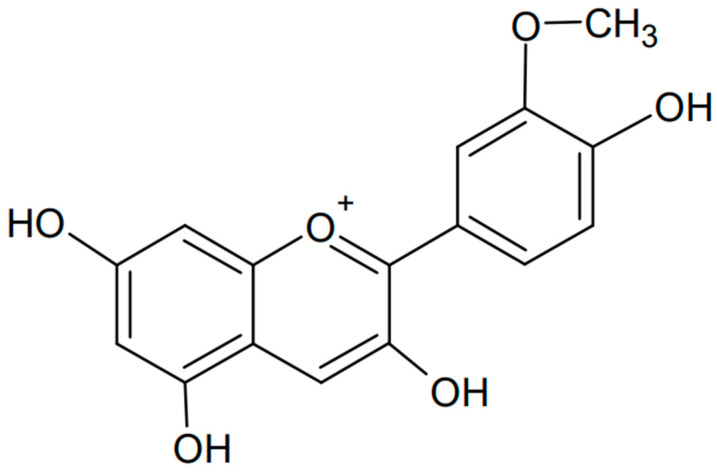
Chemical formula of peonidin (created with ChemSketch).

Another type of anthocyanin is peonidin, which is known for its red and purple hues and is found in various fruits and flowers [79,80,81]. The formula of peonidin is presented in Figure 6. Like other anthocyanins, peonidin has antioxidant properties and potential health benefits, including anti-inflammatory and anticancer effects [82]. One study showed the anticancer effects of peonidin 3-glucoside and cyanidin 3-glucoside in the highly sensitive HS578T cell line. The compounds were found to cause cell cycle arrest in the G2/M phase, reducing the levels of cyclin-dependent kinase (CDK)-1, CDK-2, cyclin B1, and cyclin E proteins. In addition, anthocyanins induced caspase-3 activation, chromatin condensation, and cell death [83]. Another study showed that peonidin 3-glucoside inhibits lung cancer metastasis via the MAPK pathway. It has been shown that anthocyanin can significantly inhibit invasion, motility, and secretion of matrix metalloproteinase (MMP)-2, MMP-9, and urokinase-type plasminogen activator (u-PA) in lung cancer cells. Phosphorylation of extracellular signal-regulated kinase (ERK)1/2, a member of the mitogen-activated protein kinase (MAPK) family involved in the upregulation of MMP and u-PA, was attenuated. Activation of activating protein-1 (AP-1) was also inhibited. The study was conducted in the H1299 cell line, and it was very important to perform complementary in vivo studies and find that peonidin inhibits lung cancer cell metastasis [84].

In addition to its anticancer properties, peonidin has antioxidant properties, helping to neutralize free radicals and protect cells from damage. This theory is supported by the results of Nas et al., in which peonidin-3-glucoside (P3G) was tested for its antioxidant properties and administered to a nematode species under various forms of stress (ultraviolet, heat, and oxidative stress). Peonidin glucoside showed free radical scavenging activity, which extended life and improved the health of the organism in the presence of UV radiation, heat, and oxidative stress, although these mechanisms may differ from its antioxidant activity [85]. This is important for deepening further research.

Other scientists analyzed twelve anthocyanins with a structure analogous to peonidin from the Chinese purple sweet potato (*Ipomoea batatas* (L.)). They were identified by LC-MS/MS. The functional properties of the anthocyanin monomers were investigated: antioxidant activity, proliferative effects on probiotics, and inhibition of harmful bacteria in vitro. Peonidin removes 1,1-diphenyl-2-picrylhydrazyl (DPPH) radicals and superoxide anions and has the potential to reduce energy consumption and Fe^2+^ chelating capacity. Anthocyanins have been shown to induce the proliferation of beneficial bacterial flora and inhibit the growth of harmful intestinal microorganisms such as *Staphylococcus aureus* and *Salmonella typhimurium*. The ability to prebiotically modulate intestinal microbiota is the basis for pharmaceutical development and the introduction of health-promoting foods to the industry [86].

### 3.3. Delphinidin

**Figure 7:**
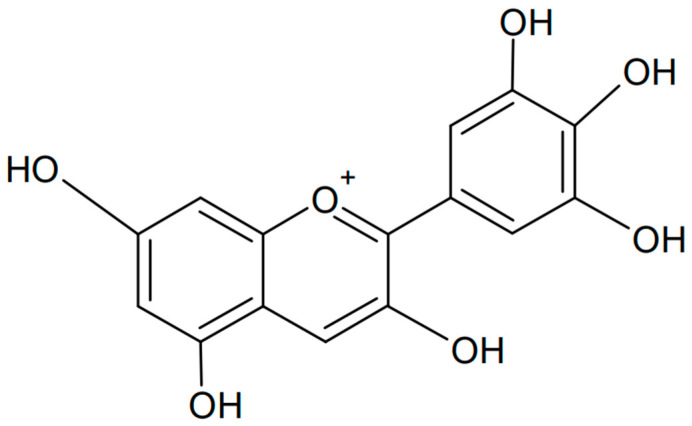
Chemical formula of delphinidin (created with ChemSketch).

Delphinidin is a polyphenolic anthocyanidin found in many plants that gives them a blue, purple, or red color, depending on the pH of the environment. The name “delphinidin” comes from the Greek word “delphinion”, which means “dolphin” (possibly for the fancied resemblance of flowers of some species to classical sculptures of dolphins), because this compound was first isolated from a plant called *Delphinium* [87]. The chemical formula of delphinidin is presented in Figure 7. Delphinidin has many beneficial health properties, including antioxidant, anti-inflammatory, and anticancer properties. It can be found in fruits such as blueberries, grapes (especially dark varieties), cranberries, and elderberries [88,89,90].

This anthocyanin is the subject of numerous scientific studies aimed at better understanding its mechanisms of action and full health potential. Much of this research focuses on its role in preventing and treating various diseases. One of the health benefits is its antioxidant effect. Delphinidin is a powerful antioxidant that helps neutralize free radicals in the body, which can protect cells from oxidative damage [91]. This is confirmed by a study carried out on pomegranate extract (*Punica granatum* L.), which contained the anthocyanins delphinidin, cyanidin, and pelargonidin. These anthocyanins exhibited scavenging activity against ·OH and O_2_·-, inhibited a Fenton reagent ·OH-generating system, possibly by chelating with ferrous ion, and inhibited H_2_O_2_-induced lipid peroxidation in rat brain homogenates [92]. Another study compared the antioxidant mechanism of delphinidin and pelargonidin using quantum chemical density functional theory and in vitro chemical antioxidant tests [93]. The geometric configuration, bond dissociation energy, and PCM (polarizable continuum) solvent model of the reaction enthalpy change were analyzed, and the reaction enthalpy change value was calculated to determine the active site (delphinidin’s C4’ site and pelargonidin’s C3 site were the free radical scavenging active sites). The results showed that the ability of delphinidin to scavenge free radicals was slightly higher than that of pelargonidin.

The antioxidant and anti-inflammatory properties of delphinidin may also support brain health, potentially reducing the risk of neurodegenerative diseases such as Alzheimer’s disease. This was demonstrated by Heysieattalab et al. [94], who showed that a high dose of delphinidin reduced acetylcholinesterase, amyloid precursor protein (APP), and amyloid beta protein (Aβ) levels in an Alzheimer’s disease model. Delphinidin treatment reduced amyloid plaque formation in rats with lesions of the nucleus basalis of Meynert (NBM). The scientific work suggested that delphinidin is a plate-like molecule sandwiched between β-plates, related to Aβ molecules, and inhibiting the formation of amyloid fibrils.

Further research shows that delphinidin can inhibit the growth of cancer cells and induce their apoptosis (programmed cell death), making it a potential agent in the fight against cancer. An experiment performed in human colon cancer HCT116 cells found that delphinidin caused a decrease in cell viability, induction of apoptosis, cleavage of PARP, activation of caspases-3, -8, and -9, an increase in Bax with a simultaneous decrease in Bcl-2, and arrest of the cell cycle in the G2/M phase. Inhibition of IKKα, phosphorylation and degradation of IκBα, phosphorylation of NF-κB/p65 at Ser536, and nuclear translocation of NF-κB/p65 were confirmed by immunoblot analysis. This suggests that delphinidin has the potential to inhibit colon cancer development [95]. In contrast, other studies performed in the human osteosarcoma-derived U2OS cell line and the breast cancer cell line MCF7 showed cytotoxic effects and induction of apoptosis via sigmoidal pathways [96,97].

In conclusion, delphinidin and delphinidin-rich fruits may be suggested as supportive treatments for a wide variety of diseases and other related disorders. These include cardiovascular diseases [98], thrombosis [99], psoriatic disease [100], hepatitis [101], and even obesity [102].

### 3.4. Petunidin

**Figure 8:**
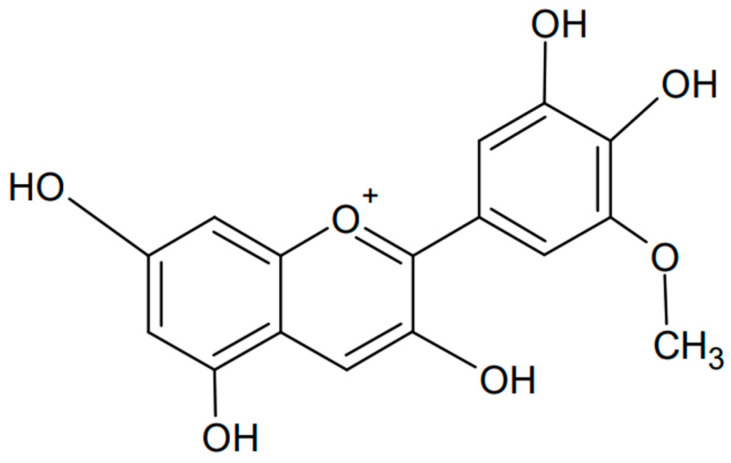
Chemical formula of petunidin (created with ChemSketch).

Petunidin is another example of an anthocyanidin, a type of plant pigment responsible for the red, purple, and blue colors of many fruits, vegetables, and flowers. Chemically known as 3,5-dihydroxy-2-(4-hydroxy-3,5-dimethoxyphenyl)chromenylium, it is found in various fruits such as grapes, berries (e.g., blackcurrants), and flowers such as petunias, from which the compound is named [103,104]. The chemical formula of petunidin is presented in Figure 8. Petunidin, like other anthocyanins, has strong antioxidant properties that help protect cells from damage caused by free radicals. This has been confirmed not only experimentally but also computationally by studying the structure and antioxidant properties of the natural food color [105]. Electron-releasing substituents, pKa, atomic charge, and bond order were examined, finding ease of hydrogen atom transfer and occurrence in the deprotonated form in blood. In addition, significant bioactivity of petunidin against nuclear receptor ligands, significant activity as a kinase inhibitor, and moderate activity as an enzyme and protease inhibitor were found. Petunidin may be a potential antioxidant used in pharmacology.

A study of the correlation of petunidin with carotenoids was also conducted using lycopene as an example and possible interactions that occur between them [106]. A model of a cardiac myofibroblast cell line (H9c2) induced by hydrogen peroxide (H_2_O_2_) was used. Petunidin synergistically interacted with lycopene, and each of them significantly protected against the loss of cell viability and activity of intracellular antioxidant enzymes (superoxide dismutase, catalase, glutathione peroxidase). The health-promoting effect is mainly based on the activation of the Nrf2 antioxidant response pathway.

In addition to its antioxidant activity, petunidin also has anti-inflammatory effects. The combination of these two properties and their mutual interpenetration are well reflected in a study on Hashimoto’s thyroiditis—a common autoimmune thyroid disease [107]. The two main pathophysiological factors of the disease are inflammation and oxidative stress. C57BL/6N mice treated with petunidin had reduced levels of TPOAb, TgAb, T3, T4, IgG, IgA, and IgM, which were accompanied by significant changes in the shape of follicles and increased lymphocyte infiltration. Anthocyanin improved thyroid dysfunction by inhibiting apoptosis and reducing ROS levels, MDA content, CD4+ levels, IFN-γ and IL-17A levels, and Th1/Th17 differentiation by regulating the NOX4/PKM2 axis.

Petunidin can also significantly improve isolated heart function in rats with anoxia/reoxygenation-induced myocardium injury [108]. It inhibits cardiomyocyte apoptosis, increases Bcl-2 protein expression, decreases NOX4 and Bax expression, and reduces cytoplasmic cytochrome c levels. Another impressive and research-proven property of petunidin is its antiproliferative effect. It has also been used to improve sRANKL-induced osteopenia in mice, increasing osteoid formation and inhibiting bone resorption. Petunidin reduced the expression of c-fos, Nfatc1, Mmp9, Ctsk, and Dc-stamp mRNA in RAW264.7 cells [109].

In studies on nematodes, it was found that petunidin glucoside significantly extended the average lifespan by 19.50%, increased motility, and reduced lipofuscin accumulation, while maintaining fertility [110]. Petunidin has many properties that may contribute to its anticancer effects, such as antioxidant, anti-inflammatory, antiproliferative, and apoptosis-inducing effects on cancer cells [111]. However, further research is needed to fully understand its potential and safety of use.

### 3.5. Malvidin

**Figure 9:**
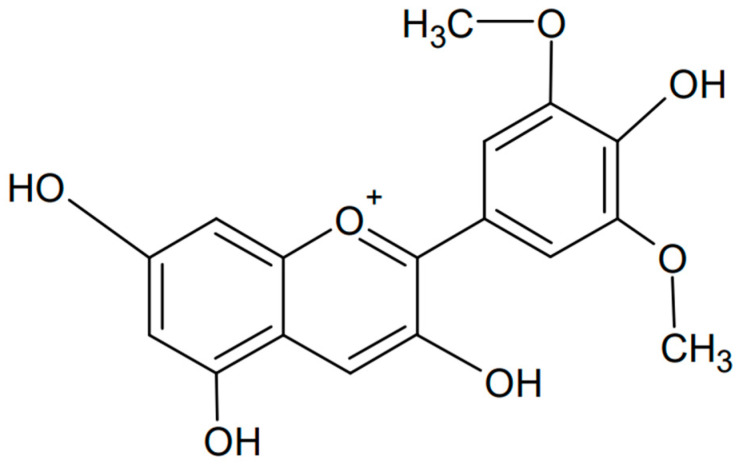
Chemical formula of malvidin (created with ChemSketch).

Malvidin is a type of anthocyanin, a compound with antioxidant properties that contribute to the color of many fruits, flowers, and vegetables. Malvidin is particularly known for imparting the blue or purple hue often found in grapes, berries, and other dark fruits [112,113,114,115]. Chemically known as 3′,5′-dimethoxy-3,4′,5,7-tetrahydroxyflavylium, it is an anthocyanidin cation whose structure is similar to delphinidin but has methyl groups attached to positions 3′ and 5′ (PubChem CID: 159287). The chemical formula of malvidin is presented in Figure 9. Like other anthocyanins, malvidin acts as an antioxidant, helping to neutralize free radicals and reduce oxidative stress. This was confirmed when malvidin glycosides found in blueberries were tested [116]. The anthocyanins reduced levels of reactive oxygen species (ROS) and xanthine oxidase-1 (XO-1) and increased superoxide dismutase (SOD) and heme oxygenase-1 (HO-1). Such properties may protect cells from oxidative degradation and offer the possibility of using malvidin as a potential functional food to prevent oxidative stress-related diseases. It has also been shown that malvidin is the most abundant polyphenol in red wine, and that its moderate consumption may impact chronic inflammatory diseases such as obesity, diabetes, high blood pressure, and cardiovascular diseases [117]. Attenuation of lipopolysaccharide-induced nuclear factor-kappa B, poly-ADP-ribose polymerase, and mitogen-activated protein kinase activation, reactive oxygen species production, and mitochondrial depolarization, along with a concomitant increase in mitogen-activated protein kinase phosphatase-1 expression and Akt activation, are examples of parameters influenced by malvidin.

The antitumor potential of malvidin is another area of interest in medicine. Its activity has been confirmed in various types of cancer, such as leukemia, colon/rectal cancer, stomach cancer, liver cancer, lung cancer, oral cancer, and breast cancer in both in vivo and in vitro studies [118]. Malvidin and its glycosides have been shown to induce apoptosis, autophagy, cell cycle arrest, suppression of cell proliferation, and prevention of metastasis by modulating different signaling pathways. For example, a study on hepatocellular carcinoma found that malvidin glucoside significantly regulated the microbial TCA cycle of the KEGG pathway by improving the expression of key proteins (porA, DLAT, aceE, PC, and OGDH) [119]. Another experiment on gastric and esophageal adenocarcinomas resulted in significant cell arrest in the G0–G1 phase of the cell cycle [120]. Other studies have highlighted the importance of the JAK/STAT-3 pathway in oral cancer, where malvidin acts as a STAT-3 inhibitor in the SCC13 oral cancer cell line, inhibiting STAT-3 phosphorylation and nuclear translocation, thereby inducing cell cycle arrest and mitochondrial-mediated apoptosis [121].

These examples confirm the importance of further scientific research into the potential therapeutic applications of malvidin due to its health benefits. 

## 4. Anti-Inflammatory Properties of Anthocyanins

Inflammation is a generalized reaction of the body that occurs as a result of the action of a damaging factor [122]. It can be a physical, mechanical, chemical, or biological factor [123]. The causes can also be divided into endogenous and exogenous. The purpose of inflammation is to mobilize the body, the cells, and mediators produced by the cells to neutralize the threat and fight it. Inflammation is characterized by redness, swelling, fever, loss of function, and pain [124]. The mechanism of inflammation is quite complex and can be divided into three main phases: initiation, propagation, and termination. In the first phase, tissue damage or infection leads to the release of inflammatory mediators (e.g., histamine, prostaglandins, leukotrienes, tumor necrosis factor (TNF), interleukin-1 (IL-1), and interleukin-6 (IL-6)). Blood vessels dilate, and their permeability increases. Immune system cells are recruited to the site of damage (e.g., leukocytes). In the next stage, immune system cells are activated and migrate: neutrophils, basophils, eosinophils, lymphocytes, and monocytes [125]. The release of additional inflammatory mediators and increased vascular permeability leads to edema. The final phase occurs after the inflammatory factor is removed. The body mobilizes reserves to repair damaged tissues, which contributes to the end of the inflammatory response and the return to homeostasis [126]. Freeing the host organism from the primary cause of inflammation protects it from the development of many diseases and illnesses. It is said that inflammation is a reaction necessary for survival [127].

Recent studies have reported that anthocyanins are becoming increasingly popular due to their numerous health benefits, including their anti-inflammatory properties. Due to their strong antioxidant properties, anthocyanins may play an important role in the treatment and prevention of inflammation [128]. In addition to studies confirming the anti-inflammatory effect of anthocyanins conducted in in vitro cell lines and in vivo animal studies, which are presented in Table 2, there are also clinical trials of the use of plant-derived compounds in humans. Although studies in humans are less numerous, there is evidence that anthocyanin consumption may be beneficial in the context of inflammatory diseases such as arthritis, heart disease, and inflammatory bowel disease.

A study conducted on a population of 2375 Framingham Heart Study Offspring Cohort participants showed that higher anthocyanin intake was inversely correlated with all twelve individual biomarkers of inflammation considered in the study [129]. Another randomized controlled trial focused on the problem of hypercholesterolemia based on the inflammatory disease atherosclerosis. Anthocyanin intake significantly reduced levels of high-sensitivity C-reactive protein (hsCRP), soluble vascular cell adhesion molecule-1 (sVCAM-1), plasma IL-1β, and LDL cholesterol compared with placebo [130]. Anthocyanins have significant potential as anti-inflammatory agents due to their antioxidant properties and ability to modulate inflammatory pathways. 

**Table 2:** Anti-inflammatory properties of anthocyanins in in vitro and in vivo studies.

Plant/Source	Compounds	Cell Line/Model	Effect	In Vitro/In Vivo	Ref.
Petunidin chloride with a purity of 99%,	petunidin (anthocyanins) and lycopene (carotenoids)	hydrogen peroxide (H_2_O_2_)-induced heart myofibroblast cell (H9c2) line model	protected against the loss of the cell viability, increased: superoxide dismutase (SOD), catalase (CAT), glutathione peroxidase (GSH-Px) induced: expression of (mRNA), protein of NAD(P)H quinone reductase (NQO1) and heme oxygenase (HO-1) of the nuclear factor erythrocyte 2-related factor 2 (Nrf2)	In vitro	[106]
*Syzygium cumini*	delphinidin 3,5-diglucoside (DDG),petunidin-3,5-diglucoside (PDG), malvidin 3,5-diglucoside (MDG)	lipopolysaccharide (LPS)-induced RAW264.7 macrophages	inhibition of nitric oxide release and pro-inflammatory mediators: mouse interleukin 6 (IL-6), mouse interleukin (IL-1β) and mouse tumor necrosis factor (TNF-α)	In vitro	[131]
*Solanum lycopersicum*	approximately 11 to over 23 different kinds of anthocyanins, including 3 anthocyanidins (delphinidin, petunidin (petunidin-3-glucoside), and malvidin)	661W a mouse photoreceptor cell line	inhibition of H_2_O_2_-induced cell death, inhibition of ROS production, suppression of apoptosis	In vitro	[132]
*Chrysobalanus icaco* L.	delphinidin-3-glucoside, cyanidin 3-glucoside, petunidin 3-glucoside, delphinidin 3-(6″-acetoyl) galactoside, delphinidin 3-(6″-oxaloyl) arabinoside, peonidin 3-glucoside, petunidin 3-(6″-acetoyl) galactoside or petunidin 3-(6″-oxaloyl) arabinoside, peonidin 3-(6″-acetoyl) glucoside, or peonidin 3-(6″-oxaloyl) arabinoside	TNF-α induced non-malignant colonic fibroblasts CCD-18Co and HT-29 colorectal adenocarcinoma cells	decreased intracellular ROS production in CCD-18Co, decreased TNF-α, IL-1β, IL-6 expression	In vitro	[133]
Purple carrots (Purple Haze) or potatoes (MacIntosh)	cyanidin-3-O-(2″-xylosyl-6″-(6‴-feruloyl-glucosyl)-galactoside and petunidin-3-O-(p-coumaroyl)-rutinoside-5-Oglucoside	Caco-2 BBe1/THP-1 co-culture cell model, gastrointestinal model	inhibited cellular inflammation, inhibited IL-8 and TNF-α secretion and expression of pro-inflammatory cytokines by blocking NF-κB, and MAPK mediated inflammatory cellular signaling cascades	In vitro	[134]
Commercial	delphinidin and petunidin	rat cardiomyocytes line H9c2	DFT calculation- the antioxidant activity from chemical mechanism	In vitro	[135]
Bilberry (*Vaccinium myrtillus* L.)	delphinidin-3-galactoside chloride, delphinidin 3- glucoside chloride, cyanidin-3-galactoside, cyanidin-3-glucoside, petunidin-3-glucoside, and cyanidin-3-arabinoside	RAW 264.7 cell line	anti-inflammatory and antioxidant activity, inhibited oxidation of linoleic acid, suppressed nitric oxide (NO) generation, pro-inflammatory cytokines iNOS, COX-2, TNF-α, and IL-6 in LPS-induced cells	In vitro	[136]
Anthocyanidins purified by HPLC (Irvine, CA, USA)	petunidin, delphinidin, cyanidin, pelargonidin, malvidin, and peonidin	human aortic smooth muscle cells (HASMCs)	inhibited PDGF-BB-induced phosphorylation of focal adhesion kinase (FAK), suppressed FAK activity by binding in an ATP, reduced HASMC migration, inhibited PDGF-BB-induced FAK phosphorylation, F-actin reduction, and FAK activity, protected against atherosclerosis	In vitro	[137]
Plant source	cyanidin (Cy), peonidin (Pn), pelargonidin (Pg), malvidin (Mv), delphinidin (Dp), and petunidin (Pt)	referenced cell lines, ulcerative colitis	reduced expression levels of TNF-α and IL-1β, reduced serum levels of IL-12 and IFN-γ, down-regulated IFN-γ, and the inflammation-associated ROS-producing enzyme myeloperoxidase (MPO), NF-κB signaling pathway, prevented increase of IL-6, and nitric oxide synthase (iNOS)	In vitro	[138]
Four varieties of beans: Negro 8025, Bayo Victoria, Pinto Durango, and Pinto Saltillo	delphinidin-3-glucoside, malvidin-3-glucoside, petunidin-3-glucoside, pelargonidin-3-glucoside, and cyanidin-3-glucoside	human intestinal cell model	antioxidant activities, inhibited lipid peroxidation, chelating capacities, deoxy-D-ribose degradation, decreased interleukin-8 (IL-8), modulated interleukin-10 (IL-10), inhibited tumor necrosis factor alpha (TNFα), NF-κβ, cyclooxygenase-2 (COX-2)	In vitro	[139]
Red clover (*Trifolium pratense*)	delphinidin-3, 5-O-diglucoside, Cyanidin-3-O-galactoside, Cyanidin-3-O-glucoside, Petunidin-3-O-galactoside, Peonidin-3-O-galactoside, Malvidin-3-O-galactoside, Petunidin-3-O-rutinoside	mouse monocyte RAW 264.7 cells	anti-inflammatory and antioxidant effects, suppressed expression of genes: TNFα, interleukin (IL)1β, inducible nitric oxide synthase (iNOS), monocyte chemoattractant protein (MCP)1, and cyclooxygenase (COX)2, stimulated intracellular reactive oxygen species (ROS), increased NADPH oxidase 1 (NOX1) and phosphorylation of p47phox, nuclear factor erythroid 2-related factor 2 (NRF2), factor kappa B (NF-kB), reduced iNOS, COX2	In vitro	[140]
Red grape skin	delphinidin 3-glucoside, Cyanidin 3-glucoside, Petunidin 3-glucoside, Peonidin 3-glucoside, Malvidin 3-glucoside, Peonidin 3-(6″-acetyl)-glucoside, Malvidin 3-(6″-acetyl)-glucoside, Delphinidin 3-(6″-coumaroyl)-glucoside, Malvidin 3-(6″-caffeoyl)-glucoside, Petunidin 3-(6″-coumaroyl)-glucoside, Peonidin 3-(6″-coumaroyl)-glucoside, Malvidin 3-(6″-coumaroyl)-glucoside	R3/1 cell line	anti-inflammatory activity, decreased NF-kb reporter and IL-1α	In vitro	[141]
Rabbiteye blueberry (*Vaccinium ashei*)	delphindin-3-galactoside, delphindin-3-glucoside, cyaniding-3-galactoside, petunidin-3-galactoside, cyaniding-3-glucoside, cyaniding-3-arabinoside, petunidin-3-glucoside, peonidin-3-galactoside, petunidin-3-arabinoside, peonidin-3-glucosidea, malvidin-3-galactoside, malvidin-3-glucoside, malvidin-3-arabinose	endothelial cell culture-HRCECs human retinal capillary endothelial cells	antioxidant and anti-inflammatory, decreased the reactive oxygen species ROS, increased the enzyme activity of catalase (CAT) and superoxide dismutase (SOD), inhibited: intercellular adhesion molecule-1 (ICAM-1), nuclear factor-kappa B (NF-κB), Akt pathway, Nox4 expression, nitric oxide (NO) levels, decreased vascular endothelial cell growth factor (VEGF)	In vitro	[142]
Commercial	cyanidin-3-O-glucoside chloride, delphinidin -3-O-glucoside cholride, pelargonidin-3-O-glucoside chloride, malvidin-3-O-glucoside chloride, peonidin-3-O-glucoside chloride, and petunidin-3-O-glucoside chloride combined with lutein	Caco-2 cell model	suppression of pro-inflammatory mediators IL-8, NO, antioxidant effects, antagonistic interaction on lipid peroxidation in a phosphatidylcholine liposome membrane with lutein	In vitro	[143]
Purple tomato (*Solanum lycopersicum* L.) line V118 and red tomato H5108 F1	petunidin-3-O-caffeoyl-rutinoside-5-O-glucoside, petunidin-3-O-(p-coumaroyl)-rutinoside-5-O-glucoside, and malvidin-3-O-(p-coumaroyl)-rutinoside-5-O-glucoside	MCF-10A breast epithelial cells, simulated gastrointestinal digestion model	anti-inflammatory effect, reduced MDA and NO production, increased GPx, SOD, protection against oxidative stress	In vitro	[144]
Extract from banana bract	malvidin, petunidin, delphinidin-3-O-galactoside, peonidin-3-O-beta-galactopyranoside, cyanidin 3-O-xylosyl-rutinoside, cyanidin 3-O-rutinoside, malvidin-3-rutinoside, pelargonidin-3-O-glucoside, delphinidin-3-O-rutinoside	LPS-stimulated murine macrophages– RAW 264.7	anti-inflammatory properties, antioxidant activity, suppressed the NO, pro-inflammatory and PGE2 production, increased the endogenous antioxidants substances level, inhibited the nuclear translocation of NF-κB	In vitro	[145]
Purple yam, *Dioscorea alata* L.	cyanidin-3,5-diglucoside, cyanidin-3-diglucoside-5-glycosides, delphinidin-3-glucose-5-rutinoside, delphinidin-3-glucoside, delphinidin-3,5-diglucoside	trinitrobenzenesulfonic acid (TNBS)-induced colitis mouse model	reduced MPO concentrations, tumor necrosis factor α, interferon γ, iNOS, decreased expression of proteins ZO-1, claudin-1, occludin, mucin-1, and mucin-2	In vitro	[146]
Commercial	cyanidin-3-glucoside, delphinidin-3-glucoside, malvidin-3-glucoside, peonidin-3-glucoside, pelargonidin-3-glucoside, petunidin-3-glucoside	human carcinogenic colon Caco-2 cells	antioxidant and anti-inflammatory effects, modulated the biomarkers: reactive oxygen species (ROS), reactive nitrogen species (RNS), pro-inflammatory cytokines and chemokines: IL-8, IL-6, IL-1β, PGE2; defensive enzymes: catalase, superoxide dismutase; intracellular signaling pathways NF-κB, mitogen-activated protein kinase	In vitro	[147]
*Gynura bicolor* DC.	cyanidin, keracyanin, kuromanin, malvidin, pelargonidin, peonidin, and petunidin	human umbilical vein endothelial (HUVE) cells	antioxidative and anti-inflammatory potentials, decreased reactive oxygen species formation, preserved glutathione content and retained glutathione peroxide and catalase activities, down regulated interleukin-6, tumor necrosis factor-alpha and prostaglandin E2 production, reduced cyclooxygenase-2 activity	In vitro	[148]
Red (RO) and yellow (YO) onion peel extracts	cyanidin-O-malonylhexoside, peonidin-O-hexoside, delphinidin-O-hexoside acetate, cyanidin-O-hexoside acetate, petunidin-O-hexoside	human adenocarcinoma cells from the MCF-7 and HT-29 cell line	suppressed NLRP3/caspase-1 signaling, decreased inflammatory cytokines, Notch-1 levels, boosted VEGF-mediated angiogenesis, reduced microbial infection, inflammation, and promoted tissue regeneration	In vitro	[149]
Plant materials: two fruits (mahaleb cherry and blackcurrant) and two vegetables (black carrot and “Sun Black” tomato)	pelargonidin, cyanidin, delphinidin, peonidin, petunidin, and malvidin and their mono-, di-, or tri-glycosides	human microvascular endothelial cell line (HMEC-1)	antioxidant effects, cardiovascular protection, expression of endothelial adhesion molecules VCAM-1 and ICAM-1	In vitro	[150]
Northern highbush blueberry extract	delphinidin, cyanidin, petunidin, peonidin, and malvidin with variation existing in the number of hydroxyl groups, methylation, location of sugar molecules	colon epithelial cell lines, NCM 356 and CCD 841 CoN	decreased in nuclear and cytoplasmic generated reactive oxygen species (ROS), increased cell viability, inhibited IL-1b, NF-jB, COX-2 expression	In vitro	[151]
Bilberry (*Vaccinium myrtillus* L.)	delphinidin, cyanidin, petunidin, peonidin and malvidin	HeLa-TLR4, THP-1, and human embryonic kidney (HEK)TLR2/HEK-TLR4 cell lines	anti-inflammatory effect, decreased tumor necrosis factor-α, interleukin (IL)-6, IL-1β expression, induced nitric oxide synthases, cyclooxygenases, nuclear factor kappa B, and Janus kinase-signal transducer and activator of transcription signaling pathways	In vitro	[152]
Grape (*Vitis vinifera* L.)-16 grape varieties	the 3-O-monoglucosides of delphinidin, cyanidin, petunidin, peonidin, and malvidin	human epithelial gastric cells (AGS)	reduced IL-8, TNFα, alleviated inflammatory processes	In vitro	[153]
Red wine extract	delphinidin, petunidin, peonidin, malvidin	murine macrophage cell line J774A.1 and Raw 264.7	prevented the carcinogenesis process, modulated IL-1β secretion, NLRP3 inflammasome pathway, increased the synthesis of NLRP3 and pro-IL-1β proteins, inhibited express the adaptor protein ASC (apoptosis-associated speck-like protein containing a CARD), inflammasome complexes	In vitro	[154]
Black soybean	three major anthocyanins (cyanidin-3-O-glucoside, delphinidin-3-O-glucoside, and petunidin3-O-glucoside) ~ about 90% of the total peak area and six minor anthocyanins cyanidin3-O-glucoside, delphinidin-3-O-glucoside, and petunidin-3-O-glucoside	3T3-L1 mouse embryo fibroblasts and RAW264.7 macrophage cells	anti-inflammatory, antidiabetic effect, decreased the production of reactive oxygen species and inflammatory mediators and cytokines (NO, MCP-1, PGE2, TNFα, and IL-6) and the release of free fatty acids, increased anti-inflammatory adiponectin secretion	In Vitro	[155]
*Vitis labrusca* extract	3-O-glucosides: peonidin, delphinidin, petunidin, and malvidin; 3-p-coumaroyl-glucosides: cyanidin, peonidin, petunidin and malvidin, and malvidin-3,5-diglucoside	Swiss mice	reduced carrageenan-induced mechanical and thermal hyperalgesia, paw edema, and neutrophil recruitment, anti-inflammatory properties	In vivo	[156]
*Vaccinium myrtillus*-bilberry	delphidin-3-O-galactoside, delphidin-3-O-glucoside, delphidin-3-O-arabinoside, cyanidin-3-O- galactoside, cyanidin-3-O-glucoside, petunidin-3-O-galactoside, cyanidin-3-O-arabinoside, petunidin-3-O-glucoside, peonidin-3-O-galactoside, petunidin-3-O-arabinoside, peonidin-3-O-glucoside, malvidin-3-O-galactoside, malvidin-3-O-glucoside, malvidin-3-O-arabinoside	mouse model	anti-inflammatory properties, decreased lipid peroxidation and mucosal injury in the ileum, directly reduced ROS, decrease of Myeloperoxidase	In vivo	[157]
Black soybean	glycosides of cyanidin, delphinidin, malvidin, pelargonidin, peonidin, and petunidin	rat model with Peyronie disease	anti-inflammatory and antifibrosis activities, decreased TGF-b1 expression, decrease activity of fibrin injections	In vivo	[158]
Substance obtained commercially	Petunidin chloride (purity ≥ 98%)	adult male Sprague–Dawley (SD) rats	improved isolated heart function, reduced oxidative stress, up-regulated Bcl-2 protein expression, down-regulated NOX4 and Bax expression, reduced the level of cytoplasmic cytochrome c	In vivo	[108]
Substance purified by HPLC	Petunidin, delphinidin, cyanidin, pelargonidin, malvidin, and peonidin	rat in vivo and rat aorta ex vivo	reduced (HASMC) human aortic smooth muscle cell migration, inhibited PDGF-BB-induced FAK phosphorylation, F-actin reduction, and FAK activity	In vivo	[137]
*Solanum lycopersicum* L.	petunidin-3-O-caffeoyl-rutinoside-5-O-glucoside, petunidin-3-O-(p-coumaroyl)-rutinoside-5-O-glucoside and malvidin-3-O-(p-coumaroyl)-rutinoside-5-O-glucoside	carrageenan-induced paw edema rat	reduced MDA and NO production, increased GPx and SOD activities in edematous tissue, inhibited paw edema formation	In vivo	[144]
Red onion (RO) and yellow (YO) onion peel extracts	cyanidin-O-malonylhexoside, peonidin-O-hexoside, delphinidin-O-hexoside acetate, cyanidin-O-hexoside acetate, petunidin-O-hexoside	rat	suppressed NLRP3/caspase-1 signaling, decreased inflammatory cytokines, Notch-1 levels, boosted VEGF-mediated angiogenesis, reduced microbial infection, inflammation, and promoted tissue regeneration	In vivo	[149]
Bilberry anthocyanin extract	delphinidin-3-galactoside, delphinidin-3-glucoside, cyanidin-3-galactoside, delphinidin-3-arabinoside, cyanidin-3-glucoside, petunidin-3-galactoside, cyanidin-3-arabinoside, petunidin-3-glucoside, peonidin-3-galactoside, petunidin-3-arabinoside, peonidin-3-glucoside, malvidin-3-galactoside, malvidin-3-glucoside, malvidin-3-arabinoside	model of phototoxicity in pigmented rabbits	reduced changes in apoptotic proteins (Bax, Bcl-2, and caspase-3), increased the levels of superoxide dismutase, glutathione peroxidase, and catalase, decreased the malondialdehyde level, inhibited the levels of pro-inflammatory cytokines and angiogenic parameters (IL-1β and VEGF)	In vivo	[159]
Commercial compounds	canidin, delphinidin, malvidin, pelargonidin and petunidin	the blood-–rain barrier in vivo and human U-87 MG cell line	anti-inflammatory, cardioprotective, anti-angiogenic, and anticarcinogenic properties, activation TGF-β Smad and non-Smad signaling pathways	In vivo	[160]
*Lycium ruthenicum* Murray	petunidin 3-O-[rhamnopyranosyl-(trans-p-coumaroyl)]-5-O-[β-D-glucopyranoside]	dextran sodium sulfate-induced colitis in mice	anti-inflammatory effects, reduction of the expression of pro-inflammatory cytokines and related mRNA: TNF-α, IL-6, IL-1β, and IFNγ, and promotion of the intestinal barrier function by histological and immunofluorescence analysis, increased proteins such as ZO-1, occludin, and claudin-1, blocked pro-inflammatory cytokines	In vivo	[161]
Portuguese blueberries (*Vaccinium corymbosum* L.)	malvidin, petunidin, peonidin, delphinidin, and cyanidin	2,4,6-trinitrobenzenesulfonic acid (TNBS)-induced colitis rat model	reduction in leukocyte infiltration, increased antioxidant defenses, downregulated nitric oxide synthase (iNOS) and cyclooxygenase-2 (COX-2), anti-inflammatory mechanism	In vivo	[162]
*Lycium ruthenicum* Murray (LR)	delphinidin-3-glu, cyanidin-3-glu, petunidin-3-glu, peonidin-3-glu, malvidin-3-glu, delphinidin, cyanidin, petunidin, pelargonidin and malvidin	rat model involving gouty arthritis induced by monosodium urate	decreased tumor necrosis factor-α (TNF-α), interleukin-1β (IL-1β), interleukin-18 (IL-18), prostaglandin E2 (PE2), cyclooxygenase-1 (COX-1) enzymes, paw volume, reduced inflammation	In vivo	[162]
Black soybean	delphinidin-3-O-glucoside, cyanidin-3-O-glucoside, petunidin-3-O-glucoside	adult male Sprague–Dawley rats–the chronic bacterial prostatitis (CBP) rat model	anti-inflammatory and antimicrobial effects, decreased bacterial growth, reduction of prostatic inflammation compared with the control group	In vivo	[163]

## 5. Anticancer Effect of Anthocyanins

In this paper, we also want to draw attention to a key problem of modern times: the increasing incidence of various cancers in the population and the potential use of anthocyanins as antiproliferative drugs. Anthocyanins, which are natural plant pigments, have strong anticancer effects, which is the subject of numerous scientific studies. Cancer is an abnormal and uncontrolled growth of cells in the body, which can lead to the formation of tumors (tissue masses) and metastases (spread of cancer cells to other parts of the body). Cells breaking away from the regulation of the cell cycle result in a cascade of unnatural multiplication and division. Cancers can be benign or malignant [164,165,166].

Cancers can have many causes, including genetic factors (inheritance of mutations in genes that increase the risk of cancer, e.g., BRCA1 and BRCA2 in the case of breast cancer) [167,168], environmental factors (exposure to carcinogens, e.g., tobacco smoke, asbestos, UV radiation) [169,170,171], lifestyle factors (a diet low in vegetables and fruits, lack of physical activity, excessive alcohol consumption, smoking) [172,173], infections (oncogenic viruses, e.g., human papillomavirus HPV, hepatitis B and C virus) [174,175], and chronic diseases (chronic inflammatory conditions, such as intestinal inflammation, may increase the risk of developing cancer). It turns out that anthocyanins have significant potential as anticancer agents due to their antioxidant properties, ability to induce apoptosis, inhibit cancer cell proliferation, inhibit angiogenesis, and reduce metastasis [176]. Studies carried out in A549 and H1299 lung cancer cells, as well as in vivo in mice, confirm the antiproliferative effects on lung cancer cells, reduction of tumor size, and suppression of cancer migration. The key proteins in these studies were MMP-2, MMP-9, COX-2, C-myc, cyclin D1, β-catenin, cyclin B, pERK, and VEGF. Apoptosis was promoted by Bcl-2 and PARP [177,178,179]. Another cancer for which anthocyanin extract was used as a supportive treatment is breast cancer. Different cell lines, such as BT474, MDA-MB-453, MDA-MB-231, A17, N202/1A, and N202/1E, confirmed the downregulation of the Akt/mTOR pathway, pro-survival Sirt1/survivin pathways, antiproliferation, apoptosis, and reduction of the metastatic markers Sp1, Sp4, and VCAM-1 [180,181,182]. Liver cancer and stomach cancer are other examples of the use of anthocyanins for anticancer activity. Among anthocyanin extracts from blueberries, blackberries, and grapes, the action of cyanidin in digestive system cancers is prominent. It induces apoptosis via the Bax, cytochrome C, caspase 3, and Akt pathways [183,184]. Another study highlights the induction of apoptosis via the p38/Fas/FasL/caspase 8 and p38/p53/Bax signaling pathways [185]. Anthocyanins have also been used to inhibit the growth and division of prostate cancer cells (22Rv1, PC-3, C4-2) [186]. In laboratory studies on cell lines of various cancers (e.g., breast, colon, prostate cancer), anthocyanins have demonstrated the ability to inhibit the growth of cancer cells and induce apoptosis. In animal studies (e.g., mice), anthocyanin supplementation led to a reduction in the size of tumors and the number of metastases. Examples of both in vitro and in vivo studies of the anticancer effects of anthocyanins are presented in Table 3.

## 6. Conclusions and Future Perspectives

The significant potential of anthocyanins as bioactive compounds with multiple health benefits is highlighted by the extensive body of research reviewed in this review. Both in vitro and in vivo studies have consistently demonstrated the antioxidant, anti-inflammatory, anticarcinogenic, and neuroprotective properties of these flavonoid pigments. The mechanistic insights gained from these studies indicate that anthocyanins exert their beneficial effects through multiple pathways. These include modulation of oxidative stress, regulation of inflammatory mediators, influence on gene expression, and interaction with various cellular signaling cascades. Importantly, while in vitro studies provide valuable insights into the molecular mechanisms of anthocyanin action, translating these findings to in vivo efficacy faces several challenges. The ultimate health effects of anthocyanins in living organisms are significantly influenced by factors such as bioavailability, metabolism, and the complex interactions within biological systems. The need for more comprehensive and integrated research approaches is highlighted by the discrepancies sometimes observed between in vitro and in vivo results. Future research directions should be focused on the conduct of long-term clinical trials to establish the efficacy and safety of anthocyanin supplementation in humans. It is also important to investigate the potential synergistic effects of anthocyanins with other phytochemicals and nutrients. Valuable insights will also be gained from elucidating the role of the gut microbiome in anthocyanin metabolism and bioactivity. Another important area for future study is the development of novel delivery systems to improve the bioavailability and stability of anthocyanins. New avenues for the application of anthocyanins in health care may be opened by exploring their potential in personalized nutrition and medicine. It is, of course, worth noting that there are many different compounds in nature that exhibit strong anticancer and anti-inflammatory effects, including quercetin, curcumin, resveratrol, sulforaphane, and lycopene. Each of these compounds has a different effect in different concentrations, which is related to a number of different factors ranging from bioavailability through synergistic effects to long-term effects.

In conclusion, further research is needed to fully understand their mechanisms of action and optimize their use in disease prevention and treatment, although current evidence strongly supports the health-promoting properties of anthocyanins. Anthocyanins promise to be valuable tools in the quest for improved human health and well-being as our understanding of these complex compounds increases. Ongoing scientific research into anthocyanins not only advances our knowledge of plant-derived bioactive compounds; it also contributes to the broader field of preventive medicine and nutritional science.

## Figures and Tables

**Table 1:** Examples of plants containing significant amounts of anthocyanins.

Source	Dominant Anthocyanins	Ref.
**Red grape (*Vitis vinifera* L.)**	Delphinidin-3-O-glucoside, Cyanindin-3-O-glucoside, Petunidin-3-O-glucoside, Peonidin-3-O-glucoside, Malvidin-3-O glucoside, Peonidin-3-O-acetylglucoside, Malvidin-3-O-acetylglucoside, Peonidin-3-O-p-coumarylglucoside, Malvidin-3-O-p-coumarylglucoside	[59]
**Currant (*Ribes nigrum* L.)**	Delphinidin-3-O-glucoside, Delphinidin-3-O-rutinoside, Cyanidin-3-O-glucoside, Cyanidin-3-O-rutinoside, Petunidin-3-O-rutinoside, Peonidin-3-O-rutinoside, Petunidin-3-O-glucoside, Pelargonidin-3-O-glucoside, Peonidin-3-O-glucoside, Pelargonidin-3-O-rutinoside	[53]
**Bilberry (*Vaccinium myrtillus* L.)**	Cyanidin-3-arabinoside, Cyanidin-3-galatoside, Cyanidin-3-glucoside, Delphinidin-3-arabinoside, Delphinidin-3-glucoside, Delphinidin-3-galatoside, Malvidin-3-arabinoside, Malvidin-3-galatoside, Malvidin-3-glucoside, Peonidin-3-arabinoside, Peonidin-3-galatoside, Peonidin-3-glucoside, Petunidin-3-arabinoside, Petunidin-3-galatoside, petunidin-3-glucoside	[60]
**Apricot (*Armeniaca Sibirica* L. Lam)**	Cyanidin 3-(4″-acetylrutinoside), Cyanidin3-(6″-acetylglucoside)-5-glucoside, Petunidin 3-galactoside, Cyanidin 3-rutinoside, Cyanidin 3-O-galactoside, Petunidin 3-rutinoside, Petunidin, Pelargonidin 3-p-coumarylglucoside, Malvidin 3-glucoside-pyruvate, Pelargonidin 3-lathyroside, Cyanidin 3-glucogalactoside, Cyanidin 3-(6-acetylgalactoside), Pelargonidin 3-arabinoside	[61]
**Barley**	Cyanidin 3-glucoside, Delphindin 3-glucoside, Petunidin 3-glucoside	[62]
***Syzygium cumini* L./*Eugenia jambolana***	Delphinidin-3,5-O-diglucoside, Cyanidin-3,5-O-diglucoside, Delphinidin-3-O-glucoside, Petunidin-3,5-O-diglucoside, Cyanidin-3-O-glucoside, Peonidin-3,5-O-diglucoside, Malvidin-3,5-O-diglucoside, Petunidin-3-O-glucoside, Malvidin-3-O-glucoside	[63]

**Table 3:** Anticancer properties of anthocyanins in vitro and in vivo studies.

Plant/Source	Compounds	Cell Line/Model	Effect		Ref.
*Vitis coignetiae Pulliat* *Meoru*	delphinidin-3,5-diglucoside, cyanidin-3,5-diglucoside, petunidin-3,5-diglucoside, delphinidin-3-glucoside, malvdin3,5-diglucoside, peonidin-3,5-diglucoside, cyanidin-3-glucoside, petunidin-3-glucoside, peonidin-3-glucoside, malvidin-3-glucoside	human hepatoma Hep3B cells	chemotherapeutic effects, antiproliferative, anti-invasive, apoptotic effects, inhibit cell growth by 75%, increased the amount of DNA fragments (sub-G1 fraction), mitochondrial dysfunction, reduction in antiapoptotic proteins (Bcl-2, xIAP, cIAP-1, and cIAP-2), inhibited migration and invasion	In vitro	[187]
*Vaccinium uliginosum* L. (*Chinese blueberry*)	delphinidin, cyanidin, petunidin, peonidin, malvidin	DLD-1 and COLO205 cells human colon and colorectal cancer cell lines	inhibited the promotion and progression of cancer cells, abolished mitogen-activated protein kinase (MAPK) pathway and activator protein-1 (AP-1), destroyed cancer cells by ROS, kappa B factor, cyclooxygenase 2 (COX-2)	In vitro	[188]
Substance obtained from Sandnes, Norway	cyanidin, delphinidin, petunidin, malvidin	human glioblastoma cell line U-87	inhibition of glioblastoma cell migration, decreased conversion of plasminogen to plasmin, decreased EGF, IGF-1, SF/HGF, TGF-b1, and VEGF, inducing cell migration	In vitro	[189]
*Eugenia jambolana* (Java plum)	delphinidin, cyanidin, petunidin, peonidin, malvidin	colon cancer cell line HCT-116, colon cancer stem cells (colon CSCs)	antiproliferative and pro-apoptotic properties, activation of caspase-glo 3/7 assay–fragmentation of DNA, increased mitochondrial protein cytochrome c	In vitro	[190]
*Cynodon dactylon* L.	delphinidin-3-O-acetylglucoside, petunidin-3-O-caffeoylglucoside-5-O-glucoside, petunidin-3-O-coumarylglucoside-5-O-glucoside, malvidin-3-O-monoglucoside, delphinidin-3-O-acetylglucoside-pyruvic acid, petunidin-3- O-acetylglucoside-5-O-glucoside and cyanidin-3,5-O-diglucoside	human breast cancer cells (MCF7), and the parasite Plasmodium falciparum	anticancer, antimalarial, antioxidant activity	In vitro	[191]
Commercial substance	cyanidin, delphinidin, peonidin, malvidin, petunidin	human glioblastoma cell	antiproliferative effect, induced death of glioblastoma cells by regulating the silent information regulator 3 (SIRT3)/p53 and PI3K/AKT/ERK pathways	In vitro	[192]
*Solanum tuberosum* L. var. *Vitelotte*	p-coumaroyl-5-glucoside-3-rhamnoglucosides of pelargonidin, cyanidin, peonidin, delphinidin, petunidin, malvidin	hematological cancer cell lines NB4 cells, MCF-7, HeLa, MDA-MB231, LNCaP and U937 cell lines	antiproliferative effects, antioxidant, antifungal, antimicrobial activities and inhibition of apoptosis, decreased number of cells in HeLa, MCF7, U937, NB4, MDA-MB231, and LNCaP lines, inhibited growth of Gram-positive and Gram-negative bacteria	In vitro	[193]
Wild blueberries	cyanidin, delphinidin, malvidin, peonidin, petunidin, and their glycosides	HepG-2 cells	anticancer ability, antiproliferative, apoptosis effect, antioxidant properties	In vitro	[194]
purchased from Dutendorfer, Vestenbergsgreuth, Germany	petunidin-3-O-glucoside	DBTRG-05MG glioblastoma cell line	antiproliferative activity, increased: Bax, decreased: Bcl-2, caspase-3 activity, Akt, p-Akt, ERK, phospho-ERK, SIRT3, and phosphorylated p53, induced cell death	In vitro	[195]
Blueberry fruits	delphinidin-3-O-glucoside, petunidin-3-O-glucoside and malvidin-3-O-glucoside	human cervical cancer HeLa cells	anticancer activities, arrested cell cycle at the G_2_/M phase, increased p53 protein expression, induced apoptosis, activated p38 MAPK/p53 signaling pathway, inhibited gene expression of NF-kB-dependent MMP-9	In vitro	[196]
*Ribes nigrum* L. fruit	delphinidin-3-0-glucoside, delphinidin-3-0-rutinoside, cyanidin-3-0-glucoside, cyanidin-3-O-rutinoside, petunidin-3-0-rutinoside, peonidin-3-O-rutinoside, petunidin-3-O-glucoside, pelargonidin-3-O-glucoside, peonidin-3-O-glucoside, pelargonidin-3-O-rutinoside, delphinidin-3-O-xyloside, cyanidin-3-0-xyloside, delphinidin-3-0-(6″-coumaroyl)glucoside cyanidin-3-O-(6″-coumaroyl)glucoside	human colorectal adenocarcinoma HT-29 cell line	inhibited growth of colon cancer cells, arrested cell cycle at the G0/G1 phase, induced apoptosis pathway by caspase 3, down-regulated matrix metalloproteinases MMP-2 and MMP-9, anticancer effect, modulation of accumulation intracellular ROS	In vitro	[197]
*Vitis* sp., *Vitis vinifera*	anthocyanin 3,5-diglucoside, delphinidin-3,5-diglucoside, cyanidin-3,5-diglucoside petunidin-3,5-diglucoside, delphinidin-3-glucoside, malvidin-3,5-diglucoside, peonidin-3,5-diglucoside petunidin-3-glucoside malvidin-3-glucoside	human prostate cancer cell lines, LNCaP cells, DU145 cells, skin cancer cell lines A431, HeLa, MCF7, HT-29, and others	induced mechanism of oxidative stress, apoptosis, cell death, regulation of Cip1/p21, caspase pathway, impairment of mitogenic signaling, increased TGF-β1, antioxidant, antimicrobial, anticancer, anti-inflammatory properties	In vitro	[198]
Potato	pelargonidin, petunidin, malvidin, cyanidin, peonidin, and delphinidin	human cancer cell lines such as human colon (HT29), liver (HepG2), cervical (HeLa), lymphoma (U937), and stomach cancer cells, A549 human lung cancer	induced apoptosis, suppressed phosphoinositide 3-kinase, Akt, NF-kappaB signaling pathway, antiproliferative activity, inhibition of ERK 1/2 phosphorylation, antioxidant, hypocholesterolemic, anti-inflammatory, antiobesity, anticancer, and antidiabetic properties	In vitro	[199]
*Armeniaca Sibirica* L. Lam (bitter apricot; kernel skins)	cyanidin 3-(4″-acetylrutinoside) cyanidin3-(6″-acetylglucoside)-5-glucoside petunidin 3-galactoside cyanidin 3-rutinoside cyanidin 3-o-galactoside petunidin 3-rutinoside petunidin pelargonidin 3-p-coumarylglucoside malvidin 3-glucosidepyruvate pelargonidin 3-latyroside cyanidin 3-glucogalactoside cyanidin 3-(6-acetylgalactoside) pelargonidin 3-arabinoside	human hepatocellular carcinoma (HepG2)	induced apoptosis, accumulation ROS, cell death mediated by caspase pathway, protein BCL-2 decreased, antimicrobial activity	In vitro	[200]
Pomegranate (*Punica granatum* L.)	cyanidin, delphinidin, malvidin, pelargonidin, pelargonidin 3-glucoside, peonidin, petunidin 3-glucoside	human breast cancer cell line AU565	antiproliferative effect, reduced tumor cell proliferation and intracellular oxygen reactive species, modulation of NFkB, COX2 phosphorylation, STAT3, and AKT, anti-inflammatory effect	In vitro	[201]
-	anthocyanidins (delphinidin, malvidin, peonidin, cyanidin, and pelargonidin)	HCT-116 colon, CSC cell line	inhibition of proliferation, induced apoptosis, activation of caspase 3, activation of caspase 7	In vitro	[190]
*Solanum nigrum*	delphinidin, cyanidin, petunidin, pelargonidin, peonidin,	MDA-MB-231, MCF-7, HepG2, SW480, MGC803, and others	anticancer, antioxidant, hepatoprotective, antiulcer, anti-inflammatory, antihyperlipidemic, antidiabetic, antibacterial, and antiseizure properties	In vitro	[202]
*Phoenix dactylifera* L.	petunidin	Caco-2 cell lines	inhibited growth of colon cancer cells, antiproliferative effect	In vitro	[203]
Blueberry fruits	delphinidin, cyanidin, petunidin, peonidin, pelargonidin, malvidin	B16-F10 melanoma cells	blocked cell cycle procession at the G0/G1 phase, inhibited proliferation, induced apoptosis, p21 and p27 regulation	In vitro	[204]
Berries, cherries, other fruits and vegetables	Petunidin	breast cancer cell lines, NSCLC cell lines (H1299 and A549)	inhibited cell growth, arrested G2/M cell cycle, stimulated apoptosis, decreased NF-κB, tumor xenograft growth modulating Wnt/ β-catenin, Notch pathways	In vitro	[179,205]
*Chrysobalanus icaco* L.	delphinidin-3-glucoside, cyanidin 3-glucoside, petunidin 3-glucoside, delphinidin 3-(6″-acetoyl) galactoside, delphinidin 3-(6″-oxaloyl) arabinoside, peonidin 3-glucoside, petunidin 3-(6″-acetoyl) galactoside or petunidin 3-(6″-oxaloyl) arabinoside, peonidin 3-(6″-acetoyl) glucoside, or peonidin 3-(6″-oxaloyl) arabinoside	TNF-α induced non-malignant colonic fibroblasts CCD-18Co and HT-29 colorectal adenocarcinoma cells	suppressed proliferation in HT-29 cells, decreased intracellular ROS production in CCD-18Co, decreased TNF-α, IL-1β, IL-6 expression	In vitro	[133]
*Vitis coignetiae* Pulliat	delphinidin-3,5-diglucoside, cyanidin-3,5-diglucoside, petunidin-3,5-diglucoside, delphinidin-3-glucoside, malvdin3,5-diglucoside, peonidin-3,5-diglucoside, cyanidin-3-glucoside, petunidin-3-glucoside, peonidin-3-glucoside, malvidin-3-glucoside	human hepatoma Hep3B cells	inhibition of cell growth, mitochondrial dysfunction, reduction of antiapoptotic proteins (Bcl-2, xIAP, cIAP-1, and cIAP-2), inhibition of the migration and invasion of Hep3B	In vitro	[187]
*Syzygium cumini* L.	malvidin, petunidin, delphinidin, cyanidin, and peonidin	human lung cancer A549 cells	antiproliferative properties, high oxygen radical absorbance capacity, high antioxidant potential, scavenging from 2,2′-azino-bis(3-ethylbenzthiazoline-6-sulphonic acid)- and 2,2-diphenyl-1-picrylhydrazyl and ferrous ion-chelating activities	In vitro	[206]
Four varieties of beans: Negro 8025, Bayo Victoria, Pinto Durango, and Pinto Saltillo	delphinidin-3-glucoside, malvidin-3-glucoside, petunidin-3-glucoside, pelargonidin-3-glucoside, and cyanidin-3-glucoside	human intestinal cell model	antioxidant activities, inhibited lipid peroxidation, chelating capacities, deoxy-D-ribose degradation, inhibited cell proliferation, decreased interleukin-8 (IL-8), modulated interleukin-10 (IL-10), inhibited tumor necrosis factor alpha (TNFa), NF-kb, cyclooxygenase-2 (COX-2)	In vitro	[139]
Lycium *ruthenicum* Murray	petunidin 3-*O*-[rhamnopyranosyl-(*trans*-*p*-coumaroyl)]-5-*O*-(*β*-*D*-glucopyranoside)	mouse model-colon cancer (nude mice)	specific anticancer effects, cell apoptosis, ferroptosis, reactive oxygen species (ROS), antioxidant effect, block the cell cycle in G0/G1 phase, inhibition cell proliferation and tumor growth	In vivo	[207]
Blueberry, bilberry and Indian blackberry (“Jamun”)	cyanidin, malvidin, peonidin, petunidin and delphinidin	nude mouse xenograft model and cell lines: nontumorigenic human bronchial epithelial cells (Beas2b) and the tumorigenic NSCLC H1299 (p53null/EGFRWT) and A549 (p53WT/EGFRWT) cells	Nontumorigenic effect, induction of cell apoptosis, suppression, inhibited migration of cells and invasion, influenced the oncogenic Notch and WNT pathways and b-catenin, c-myc, cyclin D1, cyclin B1, pERK, MMP9, and VEGF proteins, increased: Bcl2, PARP, decreased TNFa, NF-kappa B, prevented metastasis	In vivo	[179]
Fruits of *Vitis coignetiae Pulliat*	delphinidin-3,5-diglucoside, cyanidin-3,5-diglucoside, petunidin-3,5-diglucoside, delphinidin-3-glucoside, malvdin-3,5-diglucoside, peonidin-3,5- diglucoside, cyanidin-3-glucoside, petunidin-3-glucoside, peonidin-3-glucoside, malvidin-3-glucoside	Hep3B cells in a xenograft mouse model	inhibited the tumorigenicity of Hep3B cells, the activation NF-κB, suppressed intra-tumoral microvessel density, Ki67, anticancer effects, inhibition, proliferation, invasion	In vivo	[208]
*Lonicera caerulea* L.	cyanidin, delphinidin, petunidin, pelargonidin, malvidin, and peonidin	male BALB/cByJNarl mice–lung large-cell carcinoma	determined tumor growth, apoptosis, inflammation, metastasis, inhibited tumor growth; increased tumor apoptosis; decreased inflammatory cytokines: IL-1β, TNF-α, C-reactive protein, IL-6; decreased inflammation-related factors: cyclooxygenase-2 protein and nuclear factor-κB (NF-κB) mRNA; transforming growth factor-β, CD44, epidermal growth factor receptor, and vascular endothelial growth factor, expression of factors Ki67, CD45, PDL1, and CD73, decreased tumor sizes, increased tumor inhibition	In vivo	[71]
*Syzygium cumini*	delphinidin-3,5-O-diglucoside, cyanidin-3,5-O-diglucoside, delphinidin-3-O-glucoside, petunidin-3,5-O-diglucoside, cyanidin-3-O-glucoside, peonidin-3,5-O-diglucoside, malvidin-3,5-O-diglucoside, petunidin-3-O-glucoside, malvidin-3-O-glucoside	breast cancer, rat brain lipid peroxidation, Swiss mice, rabbits	inhibited apoptosis, hepatoprotective, antioxidant hypoglycemic, anti-inflammatory, antianemic, antibacterial, antiallergic, hypolipidemic, and antipyretic properties, inhibited apoptosis	In vivo	[209]
*Lycium ruthenicum* Murray	delphinidin-3,5-O-diglucoside, petunidin-3,5-O-diglucoside, malvidin-3,5-O-diglucoside	male BALB/c-nude mice, colon cancer cells	anticancer effects, reduced cell activity, blocked the cell cycle in the G0/G1 phase, induced apoptosis, ferroptosis, changes in mitochondrial morphology, increased ROS, malondialdehyde level, decreased protein expression: SLC7A11, GPX4, increased: TFR1	In vivo	[210]
